# *Cardamine occulta*, the correct species name for invasive Asian plants previously classified as *C. flexuosa*, and its occurrence in Europe

**DOI:** 10.3897/phytokeys.62.7865

**Published:** 2016-03-25

**Authors:** Karol Marhold, Marek Šlenker, Hiroshi Kudoh, Judita Zozomová-Lihová

**Affiliations:** 1 Department of Botany, Faculty of Science, Charles University, Benátská 2, CZ-128 01 Praha 2, Czech Republic; 2 Institute of Botany, Slovak Academy of Sciences, Dúbravská cesta 9, SK-845 23 Bratislava, Slovakia; 3 Center for Ecological Research, Kyoto University, Hirano 2-509-3, Otsu 520-2113, Japan

**Keywords:** Asian *Cardamine flexuosa*, Brassicaceae, Cardamine flexuosa subsp. debilis, *Cardamine hamiltonii*, *Cardamine occulta*, China, Cruciferae, Europe, invasive species, typification

## Abstract

The nomenclature of Eastern Asian populations traditionally assigned to *Cardamine flexuosa* has remained unresolved since 2006, when they were found to be distinct from the European species *Cardamine flexuosa*. Apart from the informal designation “Asian *Cardamine flexuosa*”, this taxon has also been reported under the names Cardamine flexuosa subsp. debilis or *Cardamine hamiltonii*. Here we determine its correct species name to be *Cardamine occulta* and present a nomenclatural survey of all relevant species names. A lectotype and epitype for *Cardamine occulta* and a neotype for the illegitimate name *Cardamine debilis* (replaced by Cardamine flexuosa subsp. debilis and *Cardamine hamiltonii*) are designated here. *Cardamine occulta* is a polyploid weed that most likely originated in Eastern Asia, but it has also been introduced to other continents, including Europe. Here data is presented on the first records of this invasive species in European countries. The first known record for Europe was made in Spain in 1993, and since then its occurrence has been reported from a number of European countries and regions as growing in irrigated anthropogenic habitats, such as paddy fields or flower beds, and exceptionally also in natural communities such as lake shores.

## Introduction

*Cardamine flexuosa* (Cruciferae) was described by Withering (1796) from the locality “Rookery at Edgbaston” in England. Recently, this name was lectotypified by Post et al. (2009) by the illustration (Fascicle. 4, Table no. 48, alternatively numbered no. 277) in Curtis’ *Flora Londinensis or, plates and descriptions of such plants as grow wild in the environs of London* (1781). Schulz (1903), in his monograph of the genus *Cardamine*, treated *Cardamine flexuosa* in a wide sense with a number of subspecies, varieties and formas. Out of the infraspecific taxa recognized by Schulz (1903), *Cardamine scutata* Thunb., *Cardamine fallax* (O.E. Schulz) Nakai and *Cardamine pennsylvanica* Willd. are now generally recognized as separate species. The remaining part of *Cardamine flexuosa* had until recently been treated as a single species distributed worldwide without the recognition of any infraspecific taxa (Jalas and Suominen 1994, Zhou et al. 2001, Al-Shehbaz et al. 2006).

It was not until the phylogenetic paper by Lihová et al. (2006) that it was realized that European and Eastern Asian populations traditionally treated as *Cardamine flexuosa* belong to two different taxa. Both DNA sequence and chromosome number data demonstrated that they represent two distinct evolutionary lineages. While the native European species *Cardamine flexuosa* is tetraploid (2n = 32, Marhold 1994, Kučera et al. 2005), Eastern Asian plants, informally treated by Lihová et al. as “Asian *Cardamine flexuosa*”, are octoploid (2n = 64, Lihová et al. 2006, T. Mandáková, Brno, unpublished data, Marhold et al., unpublished data, contrary to the assumed hexaploid level based on flow-cytometric evidence by Bleeker et al. 2008). Multiple hypotheses about the parentage of tetraploid European *Cardamine flexuosa* have been put forward, invoking both auto- and allopolyploidy (reviewed by Lihová et al. 2006 and Mandáková et al. 2014). Only recently, the cytogenetic approach (combining genomic *in situ* hybridization and comparative chromosome painting, CCP/GISH) provided unequivocal evidence that this taxon is an allopolyploid originating from the diploids *Cardamine amara* L. and *Cardamine hirsuta* L. (Mandáková et al. 2014). In turn, CCP/GISH (Mandáková et al., in prep.) revealed allopolyploidy also in Eastern Asian *Cardamine flexuosa* (as inferred earlier from molecular data, Lihová et al. 2006), but with a different parentage. Three distinct diploid genomes were identified within this octoploid, corresponding to *Cardamine amara*, *Cardamine parviflora* L. (or perhaps their unknown close relatives) and another, as yet unidentified taxon.

Morphological characters of Eastern Asian populations treated as *Cardamine flexuosa* and their differences from European populations are presented by a number of authors (e.g., Rosenbauer 2011, Hepenstrick and Hoffer-Massard 2014, Dirkse et al. 2015). Most of their descriptions, however, do not encompass the whole variation of the two taxa, and none consider differences from other Asian relatives, such as *Cardamine scutata*, so a thorough morphometric study of *Cardamine flexuosa* and related Eastern Asian taxa is required (Marhold et al. in prep.). These two taxa also show considerable differences in their ecological requirements. European *Cardamine flexuosa* occurs mostly in forest plant communities along wet forest roads or in various open habitats and is only seldom found as a weed in flower beds (often introduced with mulch of bark chips) or in greenhouses (Kudoh et al. 2006). Eastern Asian *Cardamine flexuosa*, by contrast, is primarily a weed of rice paddy fields, and perhaps only secondarily occurs in other open habitats (Kudoh et al. 1993, Yatsu et al. 2003). It was hypothesized by Lihová et al. (2006) that the origin and spread of this latter taxon are associated with the establishment of suitable man-made habitats (e.g. paddy fields). Based on morphology and molecular data, Lihová et al. (2006) reported Eastern Asian *Cardamine flexuosa* from Japan, China, Taiwan, Thailand, Vietnam, Australia, Canada, USA and Mexico.

As a consequence, based on their genetic divergence, different ploidy, allopolyploid origins, morphology, ecological requirements and distribution patterns, we are of the opinion that European and Eastern Asian populations previously treated as *Cardamine flexuosa* should be classified as two different taxa at the species level. The concept of two taxa is also adopted in the Flora of North America (Al-Shehbaz et al. 2010) and is followed by other authors reporting plants corresponding to Eastern Asian *Cardamine flexuosa* from different parts of the world, particularly Europe. Several names have been used for this taxon, namely Cardamine flexuosa subsp. debilis O.E. Schulz (e.g., Rankin Rodríguez and Greuter 2009, Lazzeri et al. 2013, Ardenghi and Mossini 2014, Hohla 2014a,b), *Cardamine hamiltonii* G. Don (e.g., Bomble 2014, Ardenghi et al. 2015, Dirkse et al. 2015, Hohla 2015) [both replacement names based on illegitimate *Cardamine debilis* D. Don (non *Cardamine debilis* Banks ex DC.)] and *Cardamine occulta* Hornem. (Klinkenberg 2015).

None of the above-mentioned names were properly typified or used unequivocally, which necessitated a thorough search for the correct species-level name for “Asian *Cardamine flexuosa*”. Here we present a nomenclatural survey of all relevant names and highlight the increasing number of records of “Asian *Cardamine flexuosa*” across Europe.

## Materials and methods

For the purpose of typifying names, herbarium specimens, especially types and authentic collections, were searched for in relevant herbaria (B, BM, C, E, KW, LINN, P, TI and UPS), and protologues were studied in relevant publications. Bibliographical citations in databases, such as IPNI (The International Plant Names Index; www.ipni.org), Tropicos (www.tropicos.org) and The Plant List (www.theplantlist.org), were also checked, and for species, links to IPNI LSID metadata are provided. In cases when specimen images were available online, stable identifiers for specimens (Hyam et al. 2012, Güntsch and Hagedorn 2013, Hagedorn et al. 2013; herbaria B, SAV), other permanent links (herbarium P) or links via JSTOR Global Plants (https://plants.jstor.org/; herbarium KW) are provided. In designating types of names of taxa, we strictly followed the International Code of Nomenclature for algae, fungi, and plants (McNeill et al. 2012). We also surveyed all relevant literature sources and gathered the first records of “Asian *Cardamine flexuosa*” in European countries and their larger administrative divisions.

## Results and discussion

### Nomenclature

The type status of species names corresponding to “Asian *Cardamine flexuosa*” in the sense of Lihová et al. (2006) has been determined, and justifications for their typifications are presented. *Cardamine occulta* is the oldest name applicable to populations of “Asian *Cardamine flexuosa*”.

***Cardamine occulta*** Hornem., Suppl. Hort. Bot. Hafn.: 71. 1819 (urn:lsid:ipni.org:names:280533-1:1.2) ≡ **Cardamine flexuosa var. occulta** (Hornem.) O.E.Schulz, Bot. Jahrb. Syst. 32: 479 (1903) (http://biodiversitylibrary.org/page/185332). Described from: “*Hab.* in China. C. intr. 1817”. **Lectotype (designated here, or perhaps holotype)**: *Cardamine occulta* mihi, sponte provenit in terra e China al[l]ata, ex h. b. Hafn. *Hornemann s.n.* – C! (ex herb. Hornemann, C10021749). Epitype (designated here): China, Zhejiang Province, Linhai County, Kuocang Mountains (括苍山), ditch along the road, 28°50.35'N; 120°58.90'E, 79 m, 18 April 2014, *K. Marhold CH18/12/2014, Yunpeng Zhao* 赵云鹏, *& Ming Jiang* 蒋明 – SAV! (SAV0006529 [http://ibot.sav.sk/herbarium/object/SAV0006529]).

There is a single specimen available in herbarium C originating from Hornemann’s collection that undoubtedly represents the single remnant of the original material for the name *Cardamine occulta*. As Hornemann (1819) referred to the specimen in the garden and not to the herbarium sheet, and as we cannot exclude that there was originally more than one specimen of this taxon in his collection, we designate the specimen as a lectotype of the name *Cardamine occulta* (admitting that the specimen might well represent the holotype). The plant on the type herbarium sheet was apparently grown from seeds at the Copenhagen Botanical Garden (“ex h[ortus] b[botanicus] Hafn[iensis]”). Perhaps cultivation at the garden might be the reason why the specimen cannot be reliably and unequivocally identified as “Asian *Cardamine flexuosa*” for the purposes of the precise application of the name *Cardamine occulta* to this taxon (especially considering the occurrence of a number of closely related taxa in China; Zhou et al. 2001). Therefore, in order to fix the application of the name *Cardamine occulta*, we designate here an epitype of this name from a cytogenetically investigated population from Eastern China with a known chromosome number (2n = 64; Mandáková et al., in prep.).

= ***Cardamine debilis*** D. Don, Prodr. Fl. Nepal. 201. 1825 [26 Jan-1 Feb 1825], (urn:lsid:ipni.org:names:280260-1:1.3; http://biodiversitylibrary.org/page/393098), nom illeg., non Banks ex DC. Syst. Nat. 2: 265. 1821 [late May 1821] (urn:lsid:ipni.org:names:280259-1:1.4; http://biodiversitylibrary.org/page/39512107). Described from: “*Hab.* in Nepaliâ ad Narainhetty. *Hamilton*.” **Neotype (designated here)**: [India, West Bengal] Botanical Garden Darjeeling, weed, 18. 6. 1959, *Lövkvist C-336-3* – UPS! (GUID UPS:BOT:V-194865) ≡ ***Cardamine hamiltonii*** G. Don, Gen. Hist. 1: 167. 1831 [early Aug 1831] (urn:lsid:ipni.org:names:280357-1:1.2.2.1.1.1; http://biodiversitylibrary.org/page/389972) ≡ **Cardamine flexuosa subsp. debilis** O.E. Schulz, Bot. Jahrb. Syst. 32: 478. 1903 (http://biodiversitylibrary.org/page/185331).

The name *Cardamine debilis* D. Don is based on data in the manuscript of Francis Buchanan-Hamilton (referred to as “Hamilton MSS”; Don 1825: 201), and it is unclear whether D. Don studied any specimen collected by Buchanan-Hamilton. Although Hara and Williams (1979) mentioned the type of *Cardamine debilis* [when indicating localities of Cardamine scutata subsp. flexuosa (With.) Hara in Nepal], in Shrestha and Press (2000), the type specimen is listed as “not found”. In any case, a thorough search in the herbaria BM, E, LINN-Smith (Roy Vickery, John Edmondson, Mark Watson, personal communication) did not reveal any original material of this name. There is a specimen corresponding to the description of *Cardamine debilis* D. Don and to “Asian *Cardamine flexuosa*”, collected in the neighbouring area of West Bengal, with a chromosome number counted by B. Lövkvist (2n = 64, unpublished data, deposited at UPS). This specimen is selected here as a neotype to fix the application of the name.

= ***Cardamine brachycarpa*** Franch., Bull. Soc. Bot. France 26: 83. 1879, nom. illeg. (urn:lsid:ipni.org:names:280196-1:1.4; http://biodiversitylibrary.org/page/260368), non Opiz, Naturalientausch 11: 411. 1826 (urn:lsid:ipni.org:names:280195-1:1.3). Described from: [JAPAN] “Insul. Nippon, prov. Etchigo, circa Niigata, secus vias humidas (R. P. Faurie)”. Lectotype (designated by Marhold et al. 2015: 11): [JAPAN, Prefecture Niigata], “Nippon, Niigata, secus vias, *[U. J.] Faurie 23*” – P! (P00747512 [http://coldb.mnhn.fr/catalognumber/mnhn/p/p00747512]); Isolectotype – P! (P00747513 [http://coldb.mnhn.fr/catalognumber/mnhn/p/p00747513]) ≡ ***Cardamine koshiensis*** Koidz., Fl. Symb. Orient.-Asiat. 43. 1930 (urn:lsid:ipni.org:names:280422-1:1.2.1.2).

= ***Cardamine arisanensis*** Hayata, Icon. Pl. Formosan. 3: 20. 1913 [25 Dec 1913] (urn:lsid:ipni.org:names:280161-1:1.3). Described from: “In Monte Morrison, ad 10000-11000 ped. alt., leg. T.Kawakami et U.Mori, 1906, Oct. (No.2252); in Montibus Centralibus, Feb. 1908”. Lectotype (Ohwi 1934: 50, see also Al-Shehbaz and Peng 2000: 237): [TAIWAN] “Kagi, Arisan (Chiayi, Alishan), Taiwan Sotoku-fu, Industry Bureau, Plant Specimens, no. 3631, 25 March 1908, *T. Kawakami & S. Mori s.n.*” (TI) ≡ ***Barbarea arisanense*** (Hayata) S.S.Ying, Alp. Pl. Taiwan in Color 2: 170. 1978.

= ***Cardamine autumnalis*** Koidz. Bot. Mag. (Tokyo) 43: 404. 1929 (urn:lsid:ipni.org:names:280169-1:1.3) – Described from: “Nippon: Yokosuka (1g. Wichura, Oct. 18, 1860) Mus. Bot. Berol.-Dahlem”. Holotype: “Japan, Jokohama, 19. [sic!] 10. 1860, *[M. E.] Wichura 1064 [1069?]*” B! (B 10 0241388 [http://herbarium.bgbm.org/object/B100241388]).

The species *Cardamine autumnalis* was described with a reference to “Cardamine flexuosa ssp. debilis Schultz (pro. parte) in Engl. Bot. Jahrb. 32. (1903) s. 479, (quoad specim. ex Yokoska)”. Indeed, there is a specimen marked “Japonia: … pr. Jokohama leg. Wichura 1860” referred to by Schulz (1903: 479) as Cardamine flexuosa subsp.debilis deposited in B. The specimen bears a revision label by Schulz with the name “Cardamine flexuosa With. subspec. debilis Don var. occulta (Hornem.) O. E. Sch.”, dated 25. 4. 1902. Although this specimen was identified by Schulz as var. occulta, it should be noted that there is no specimen referred to by Schulz (1903: 480) identified as Cardamine flexuosa subsp. debilis var. occulta from Japan.

The usual life cycle of *Cardamine occulta* in Eastern Asian rice fields includes flowering in early spring before rice is planted and the fields are flooded by water. Nevertheless, there are also exceptions such as the nomenclatural type of the name *Cardamine autumnalis*, which represents an autumn-flowering plant of *Cardamine occulta*. Kudoh et al. (1993: fig. 8) reported such plants from paddy fields in the autumns of years in which rice was not cultivated (no water flooding during summer).

‒ **Cardamine aff. flexuosa** sensu I. Thomps., Flora of Victoria 3: 434–442. 1996.

There are two other names at the species level that are potentially applicable to “Asian *Cardamine flexuosa*”, namely:

*Cardamine nasturtioides* D.Don, Prodr. Fl. Nepal.: 201. 1825. [26 Jan-1 Feb 1825] (urn:lsid:ipni.org:names:280509-1:1.3; http://biodiversitylibrary.org/page/393098) – Described from: “*Hab.* in Nepaliâ. *Hamilton*.”

*Cardamine decurrens* (Blume) Zoll. et Moritzi in Moritzi, Syst. Verz.: 35. 1846 (urn:lsid:ipni.org:names:280262-1:1.3.2.2; http://reader.digitale-sammlungen.de/de/fs1/object/display/bsb10302557_00051.html) ≡ *Pteroneurum decurrens* Blume, Bijdr. Fl. Ned. Ind. 2: 51. 1825 [12 Jun-2 Jul 1825] (urn:lsid:ipni.org:names:288262-1:1.1.2.2.1.2; http://biodiversitylibrary.org/page/428177). – Described from: “in altis paludosis montis Burangrang Provinciae Krawang.”

The location of the original material of these two names is as yet unknown, and it remains to be ascertained whether they are synonyms of *Cardamine occulta* or represent other taxa. In any case, both these names are later than *Cardamine occulta*, which has priority among all species names applicable to “Asian *Cardamine flexuosa*”.

The name *Cardamine zollingeri* Turcz. was sometimes considered to be a synonym of *Cardamine flexuosa* in a wide sense (e.g., Zhou et al. 2001, Al-Shehbaz et al. 2006, Al-Shehbaz and Watson 2012) or of Cardamine flexuosa subsp. debilis (Schulz 1903: 479). Nevertheless, it is morphologically different from both *Cardamine flexuosa* and *Cardamine occulta* in the circumscriptions presented here and likely represents a separate taxon that requires further study:

*Cardamine zollingeri* Turcz., Bull. Soc. Imp. Naturalistes Moscou 27(2): 294. 1854 (urn:lsid:ipni.org:names:280762-1:1.3) ≡ *Nasturtium obliquum* Zoll. & Moritzi, Natuur- Geneesk. Arch. Ned.-Indië 2: 580. 1845 (urn:lsid:ipni.org:names:287528-1:1.4; https://archive.org/stream/natuurengeneesku02bata#page/580/mode/2up) – Described from: “[Java] *Nasturtium obliquum* Z. et M. Herb. N. 2211 … Legi in arenosis et glareosis vulcanicis ad fluviorum ripas e. g. prope *Trawas* prov. *Modjokerto* VIII.1844. p. m. 3000’ s. m.” **Lectotype (designated here)**: [INDONESIA, Java], “Planta Javanica a cl. Zolliger lecta no. 2211” *Zollinger 2211* KW! (KW001000851 [https://plants.jstor.org/stable/10.5555/al.ap.specimen.kw001000851]); Isolectotype: P! (P00747614 [http://coldb.mnhn.fr/catalognumber/mnhn/p/p00747614]).

### Occurrence of *Cardamine occulta* in Europe

*Cardamine occulta* most likely originated in Eastern Asia. It is unclear whether it naturally occurs or ever occurred in any natural plant community. The localities that we know from Japan and Eastern China and which are referred to on herbarium specimens represent solely man-made habitats, most often rice paddies, orchards or various other kinds of synanthropic vegetation. This is why we (Lihová et al. 2006) hypothesized that the origin and spread of this polyploid species might have been connected with the occurrence of man-made habitats.

As stated above, Lihová et al. (2006) reported plants corresponding to *Cardamine occulta* from Japan, China, Taiwan, Thailand, Vietnam, Australia, Canada, USA and Mexico. Other previously published data corresponding to *Cardamine occulta* than those that were referred to by Lihová et al. (2006) were the report of *Cardamine debilis* D. Don from North America as an introduced weed (Rollins 1993) and Cardamine aff. flexuosa from Australia (Thompson 1996). Subsequently, this taxon was published also for Cuba (Rankin Rodríguez and Greuter 2009, as Cardamine flexuosa subsp. debilis).

**Figure 1.**
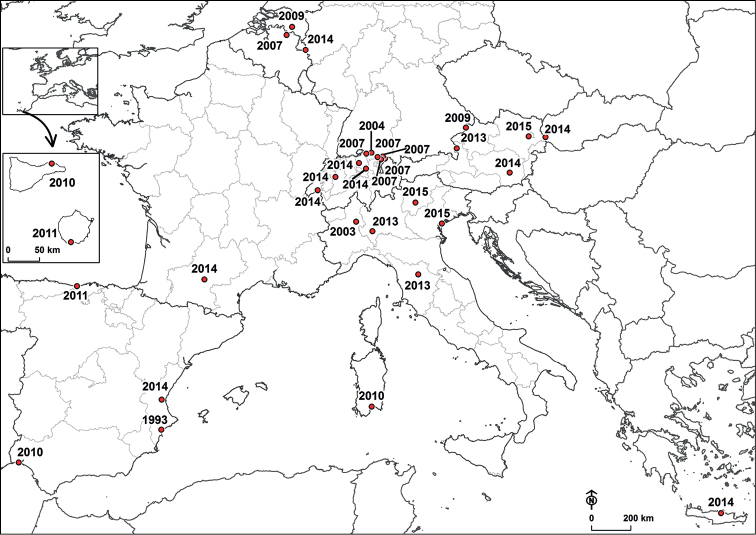
Localities of the first occurrences of *Cardamine occulta* Hornem. for European countries and their administrative divisions. The year of the first occurrence at each locality is given. The inset shows Tenerife and Gran Canaria of the Canary Islands.

When Lihová et al. (2006) suggested that European and Asian *Cardamine flexuosa* should be treated as separate taxa, no record corresponding to Asian *Cardamine flexuosa* plants was known from the European territory. Nevertheless, a number of records from Europe have been published since 2007, and we can trace the spreading of this invasive plant throughout the continent (see Table 1, Fig. 1). To the best of our knowledge, the earliest record of *Cardamine occulta* from Europe dates back to 1993, when this species was collected in the Spanish province of Alicante and originally identified as *Cardamine flexuosa*. Its true taxonomic identity was, however, clarified much later (Crespo et al. 2013). In 2007 the first author of this paper received for identification a specimen collected in 2003 in a rice field ditch in the province of Piedmont, Italy (Vercelli, Arborio) by Michel Desfayes (Fully, Switzerland). This specimen undoubtedly belongs to *Cardamine occulta* and might have been introduced together with rice from Eastern Asia. From the same broad locality, the occurrence of this taxon was reported by Thomas Götz (a specimen collected in 2005, published by Dienst 2007) and more recently by Verloove and Ardenghi (2015; as *Cardamine hamiltonii*).

**Table 1.** First records of *Cardamine occulta* Hornem. for European countries and their administrative divisions (if multiple records for a given region are dated to the same time, one representative is chosen). Information in square brackets was derived by the authors of the present paper. The records were reported under (1) *Cardamine flexuosa* auct. non With. (Asian *Cardamine flexuosa*), (2) *Cardamine flexuosa* auct. non With., (3) Cardamine flexuosa subsp. debilis O.E. Schulz, (4) *Cardamine hamiltonii* G. Don, and (5) *Cardamine occulta* Hornem.

Country	Admin. division	Year	Locality	Reported by (Reported as)
Austria	Vorarlberg	2007	Lake Constance, [Bregenz, shore of the lake, 47°30'N; 9°44'E], 2007	Bleeker et al. 2008 (2)
Austria	Upper Austria	2009	Schärding, Stadtplatz square, in flower pots and between cobblestones (7546/2), ca. 320 m, [48°27.41'N; 13°25.9'E], 7.6.2009, *M. Hohla* (LI 100238232)	Hohla 2012 (3)
Austria	Salzburg	2013	Salzburg City, Liefering, Oberer Bonau-weg Street, in the nursery as a weed, ca. 410 m (8144/3) [47°49.38'N; 13°0.78'E], 23.8.2013, *P. Pilsl* (Herbarium Pilsl), conf. M. Hohla	Hohla 2015 (4)
Austria	Styria	2014	Graz, Jakominiplatz square, in flower beds (8958/2), [47°4.05'N; 15°26.5'E], 27. 09. 2014, *M. Hohla* (LI)	Hohla 2014b (3)
Austria	Vienna	2015	Vienna, West Railway station (Westbahnhof), ca. 210 m, (7864/1) [48°11.53'N; 16°18.76'E], 8.12.2015, M. Hohla (LI).	Hohla 2015 (4)
Belgium		2007	Antwerp, Mol, [Lostraat st., cemetery], 51°12.05'N; 5°12.78'E, 29. 03. 2007, *R. Barendse* (observation)	http://waarnemingen.be/waarneming/view/45438666 (4)
France	Midi-Pyrénées	2014	Toulouse, [Square Charles de Gaulle square], urban vegetation, 43°36.28'N; 1°26.7'E, 12. 04. 2014, *E. Slootweg* (observation)	http://observation.org/waarneming/view/83277183 (4)
Germany	Baden-Württemberg	2004	Lake Constance, Reichenau, Reichenauer Damm dam, [47°41.2'N; 9°6'E], spring 2004, *W. Ostendorp, M. Dienst & E. Klein*	Dienst 2007 (2)
Germany	Bavaria	2007	Lake Constance, [Wasserburg, shore of lake, 47°34'N; 9°38'E], 2007	Bleeker et al. 2008 (1)
Germany	North Rhine-Westphalia	2014	Aachen, Soers, Garden Center (5202/21), [50°46'N; 6°5'E], 14. 03. 2014, *F. W. Bomble & S. Bomble*	Bomble 2014 (4)
Greece		2014	Crete, Nomos of Iraklion, Eparchia of Temenos, 1821 Street, near entrance of the “El Greco Hotel”, edge of flower bed with a cultivated tree, 35°20.28'N; 25°7.96'E, 17. 06. 2014, *N. M. G. Ardenghi & P. Cauzzi* (MSNM)	Ardenghi et al. 2015 (4)
Italy	Piedmont	2003	Prov. Vercelli, Arborio [45°29.6'N; 8°24'E], 25. 08. 2003, *M. Desfayes* (SAV)	M. Desfayes, unpubl. data
Italy	Sardinia	2010	Cagliari, near the building of the Department of Botany at Viale Sant’Ignazio da Laconi, 56 m, 39°13.3'N; 9°6.7'E, 03. 2012, *V. Lazzeri*	Lazzeri et al. 2013 (3)
Italy	Lombardy	2013	Pavia, Piazzale della Stazione square, public flowerbed, 45°11.3'N; 9°8.68'E, 11.12.2013, *N. M. G. Ardenghi* (MSNM)	Ardenghi and Mossini 2014 (3)
Italy	Tuscany	2013	Florence, W side of Piazza di Santa Maria Novella square, public flower bed, 43°46.41'N; 11°14.94'E, 09. 12. 2013, *N. M. G. Ardenghi & S. Mossini* (MSNM)	Ardenghi and Mossini 2014 (3)
Italy	Trentino-South Tirol	2015	Trento, Corso del Lavoro e della Scienza, 191 m, [46° 3.57'N; 11°6.95'E], 20. 11. 2015, *V. Lazzeri* (FI)	Lazzeri and Marhold 2016 (5)
Italy	Veneto	2015	Venice, [Campo San Maurizio], 45°25.97'N; 12°19.90'E, 11. 09. 2015, *W. Meijer* (observation)	http://observation.org/waarneming/view/110617765 (4)
Slovakia		2014	Bratislava, Brnianska street, flower pot with a shrub at restaurant Patrónsky pivovar, 320 m, 48°9.96'N; 17°4.84'E, 10. 06. 2014, *K. Marhold* (SAV!, SAV0006528; http://ibot.sav.sk/herbarium/object/SAV0006528)	K. Marhold, unpubl. data
Spain	Valencia, Alicante	1993	San Vicente del Raspeig, Partida Canastell, flower pot, (UTM 30SYH1455), 170 m, [38°24'N; 0°32'W], *J.C. Cristóbal* (ABH 5166)	Crespo et al. 2013 (3)
Spain	Canary Islands, Tenerife	2010	Bajamar, TF-13 road, close to Barranco Perdomo, Pelargonium plantation in roundabout, [28°32.8'N; 16°20.9'W], 15. 09. 2010, *F. Verloove 8433* (ORT 41743)	Verloove and Reyes-Betancort 2011 (2)
Spain	Andalusia, Huelva	2010	Nuevo Portil, golf course (UTM 29SPB7220), [37°12.8'N; 7°4'W], 11. 08. 2010, *E. Sánchez Gullón* (priv. herb. ESG 263; dupl. BR)	Verloove and Gullón 2012 (3)
Spain	Canary Islands, Gran Canaria	2011	San Agustin, Las Burras, close to the beach, irrigated lawn, [27°46.1'N; 15°32.5'W], 06. 11. 2011, *F. Verloove 9215* (LPA)	Verloove 2013 (3)
Spain	Cantabria	2011	San Vicente de la Barquera, 43°22.9'N; 4°23.9'W, 09. 06. 2011, M. Lysák (SAV!, SAV0006530, SAV0006531; http://ibot.sav.sk/herbarium/object/SAV0006530, http://ibot.sav.sk/herbarium/object/SAV0006531)	M. Lysák, unpubl. data
Spain	Valencia	2014	Valencia, Quart de Poblet, Mas de les Fites, 96 m, gardens of Centro para la Investigación y Experimentación Forestal de la Generalitat Valenciana (UTM 30SYJ134726) [39°28.44'N; 0°31.25'W], 19. 08. 2014, *C.J. Mansanet, P.P. Ferrer & E. Laguna* (VAL 222275)	Mansanet-Salvador et al. 2015 (3)
Switzerland	Schaffhausen	2007	Lake Constance, [Stein am Rhein, shore of lake, 47°39.4'N; 8°52'E], 2007	Bleeker et al. 2008 (1)
Switzerland	St. Gallen	2007	Lake Constance, [Staad, shore of the lake, 47°29'N; 9°32'E], 2007	Bleeker et al. 2008 (1)
Switzerland	Thurgau	2007	Lake Constance, [Salmsach, shore of the lake, 47°33'N; 9°22.8'E], 2007	Bleeker et al. 2008 (1)
Switzerland	Bern	2014	Bern, 598751/199269, flower pots, [46°56'N; 7°27'E], 2014	Hepenstrick and Hoffer-Massard 2014 (3)
Switzerland	Schwyz	2014	Lachen, 707088/227808, between cobblestone, 2014, [47°11'N; 8°51'E], 2014	Hepenstrick and Hoffer-Massard 2014 (3)
Switzerland	Vaud	2014	Lausanne, Av. de Florimont, 538763/152550, between paving stones, [46°30.9'N; 6°38.3'E], 2014	Hepenstrick and Hoffer-Massard 2014 (3)
Switzerland	Zürich	2014	Zürich, 681596/248874, gravel, [47°22'N; 8°32'E], 2014	Hepenstrick and Hoffer-Massard 2014 (3)
The Netherlands		2009	North Brabant, Eindhoven, [51°26'N; 5°28'E], 2009, *R. Barendse*	Dirkse et al. 2015 (4)

The third spot in Europe where *Cardamine occulta* was reported from are the shores of Lake Constance (Bodensee) in Germany. In spring 2004, an unknown *Cardamine* species was detected there at the Reichenau dam (observed by W. Ostendorp, M. Dienst and E. Klein; Dienst 2007). The identity of these plants was confirmed by DNA sequencing (Bleeker et al. 2008). Until 2007, 95 locations on the shores of Lake Constance had been known. Localities were found around the lake in Germany (Baden-Württemberg and Bavaria), Austria (Vorarlberg) and Switzerland (cantons Schaffhausen, Thurgau, and St. Gallen; Bleeker et al. 2008). Bleeker et al. (2008) noted that *Cardamine occulta* was more frequent on fine-grained and nutrient-rich sediments than on nutrient-poor gravel shores. It is likely that this species may change the community structure of ephemeral vegetation on bare and organic sediments.

*Cardamine occulta* was later reported also from continental Spain, the Canary Islands, France, parts of Germany, Switzerland and Austria other than the shores of Lake Constance, from Belgium, the Netherlands, Slovakia, and Crete (Table 1). It is nevertheless likely that the species is currently present, but still overlooked, also in other European countries. It should be noted that most records mentioned in Table 1 refer to urban vegetation. *Cardamine occulta* grows in flower beds and pots, at the edges of roads, among cobblestones or paving stones, or on pavements, often in irrigated places. In most cases, it was apparently introduced as a weed, often with mulch, from plant nurseries where it finds appropriate growing conditions (as reported from North America by Post et al. 2011). However, the species was also found in rice fields in northern Italy, where it was most likely introduced with rice from Eastern Asia. There are only a few known occurrences of *Cardamine occulta* in European natural plant communities, and it seems that such reports are restricted to the surroundings of Lake Constance. Bleeker et al. (2008) hypothesized that this species might have been introduced to the lake from rice fields of northern Italy by migrating birds or directly from Japan by tourists.

For most of the countries and administrative divisions presented in Table 1, only one or few localities of *Cardamine occulta* are known. There are numerous observational records of *Cardamine occulta* from the Netherlands and Belgium in the databases presented at observation.org, waarneming.nl and waarnemingen.be (referred to as *Cardamine hamiltonii*), perhaps because botanists in these countries were encouraged to searched for it. Nevertheless, there are no voucher specimens documenting these data, and some of them are not even documented by photographs. According to the photographic documentation, some records are apparently based on misidentifications of *Cardamine hirsuta* and tetraploid *Cardamine flexuosa*. A number of photographic records document juvenile plants that are hard to identify reliably. For future mapping of the distribution of *Cardamine occulta*, all records should be documented by vouchers deposited in public herbaria.

It is apparent that, unlike European *Cardamine flexuosa*, *Cardamine occulta* represents an invasive species that is quickly spreading from its area of origin in Eastern Asia to other continents. The characteristics of seed dormancy and germination of *Cardamine occulta* are likely to enhance its invasiveness, especially in wet and occasionally submerged habitats. It has been reported that seeds of *Cardamine occulta* can survive both in dry and submerged conditions for more than three months (Yatsu et al. 2003). The combination of seed dormancy in dry soil and dormancy release by submergence (Yatsu et al. 2003) is likely to enhance the transportation of *Cardamine occulta* seeds with soils and the establishment of invasive populations in seasonally submerged habitats such as paddy field, dams or lake shores and in regularly irrigated flower beds and other urban habitats. Diploid *Cardamine hirsuta* is in fact another example of the invasive potential of *Cardamine* species. This species originated in Europe and is now widely distributed on all continents, particularly in drier conditions. The speed of its spreading can be illustrated on the example of the Japanese archipelago. While the first record of this species for Japan dates to 1974 (Kudoh et al. 1992), already in 2006 it became a common roadside weed across most of Honshu Island, the main island of Japan, and was spreading also to Kyushu and Hokkaido Islands (Yatsu et al. 2003, Kudoh et al. 2007).
